# Nearby night lighting, rather than sky glow, is associated with habitat selection by a top predator in human-dominated landscapes

**DOI:** 10.1098/rstb.2022.0370

**Published:** 2023-12-18

**Authors:** Rafael Barrientos, Winston Vickers, Travis Longcore, Eric S. Abelson, Justin Dellinger, David P. Waetjen, Guillermo Fandos, Fraser M. Shilling

**Affiliations:** ^1^ Road Ecology Lab, Department of Biodiversity Ecology and Evolution, Faculty of Biological Sciences, Universidad Complutense de Madrid, José Antonio Novais 12, 28040 Madrid, Spain; ^2^ Wildlife Health Center, University of California, 1089 Veterinary Medicine Dr, Davis, CA 95616, USA; ^3^ Institute of the Environment and Sustainability, University of California Los Angeles, Los Angeles, CA 90095, USA; ^4^ Department of Integrative Biology, University of Texas Austin, Austin, TX 78705, USA; ^5^ Large Carnivore Section, Wyoming Game and Fish Department, 260 Buena Vista Dr., Lander, WY 82520, USA; ^6^ Road Ecology Center, Institute of Transportation Studies, University of California, Davis, CA 95616, USA; ^7^ Department of Biodiversity Ecology and Evolution, Faculty of Biological Sciences, Universidad Complutense de Madrid, José Antonio Novais 12, 28040 Madrid, Spain

**Keywords:** ALAN, home-range, natural illumination, *Puma concolor*, visible and infrared imaging radiometer suite, zenith brightness

## Abstract

Artificial light at night (ALAN) is increasing in extent and intensity across the globe. It has been shown to interfere with animal sensory systems, orientation and distribution, with the potential to cause significant ecological impacts. We analysed the locations of 102 mountain lions (*Puma concolor*) in a light-polluted region in California. We modelled their distribution relative to environmental and human-disturbance variables, including upward radiance (nearby lights), zenith brightness (sky glow) and natural illumination from moonlight. We found that mountain lion probability of presence was highly related to upward radiance, that is, related to lights within approximately 500 m. Despite a general pattern of avoidance of locations with high upward radiance, there were large differences in degree of avoidance among individuals. The amount of light from artificial sky glow was not influential when included together with upward radiance in the models, and illumination from moonlight was not influential at all. Our results suggest that changes in visibility associated with lunar cycles and sky glow are less important for mountain lions in their selection of light landscapes than avoiding potential interactions with humans represented by the presence of nearby lights on the ground.

This article is part of the theme issue ‘Light pollution in complex ecological systems’.

## 1. Introduction

Globally, human development continues to expand, challenging the ability of organisms to cope with anthropogenic pressures such as land use changes (i.e. agriculture expansion, urban development), development of infrastructure networks, and night-time light pollution [1–3]. Pollution from artificial light at night (ALAN) is occurring on a global scale, similar to well-recognized forces of environmental change such as land cover change [2]. Among the heterogeneous responses of biodiversity to ALAN are the advance of spring leaf budding in deciduous trees [4], inhibition of mating in insects under artificial lights [5], wildlife shifts to darker/brighter areas where perceived predation risk is lower [6,7], changes in reproductive timing or success of birds in response to light leading to phenological mismatches and lower fitness [8], and avoidance of lit wildlife crossings by mammals creating a barrier effect for a linear infrastructure [9]. A recent synthetic review suggested more studies are needed to test for ALAN effects at lower intensities (e.g. from skyglow), which many organisms experience throughout large areas worldwide [6]. Following this line of thought, we felt it would be worth investigating the response of organisms to the effects of artificial light at night, versus natural illumination from moonlight.

Roads are an important form of encroachment into natural landscapes [10,11], and ALAN follows roads, compounding the impacts of roads across the few uninfluenced ecosystems [2,12,13]. Whereas roads affect virtually all terrestrial species, large predators are particularly at risk due to their large territory requirements, small population sizes and low reproductive rates [14]. Even habitat generalists like mountain lions (also known as pumas, cougars or panthers; *Puma concolor*), which are continuously distributed from Canada to Patagonia [15], can be at risk. Mountain lion habitat selection is very flexible, leading them to inhabit diverse areas, from high mountains to marshlands, from deserts to tropical forests [16–18]. Some investigators have suggested that mountain lions avoid residential development and human infrastructure, like roads [18–20]. However, like other large carnivores in North America and Europe [21,22], habitat restoration and conservation actions have facilitated mountain lion population persistence and even recoveries of their previously occupied range in increasingly human-dominated landscapes [15,23]. This inevitably leads to increases in conflict with their new neighbours, humans [19,24]. It is largely unknown, however, to what extent different disturbance sources affect mountain lion and other wildlife population dynamics, impacts that ultimately can affect their population persistence [25,26]. Large carnivores patrol huge territories because the resources on which they depend are heterogeneous both in space and time [e.g. 16,19,27]. In these movements, these top predators usually encounter landscapes polluted by artificial lights and crossed by roads.

Mountain lions are likely to respond differently to light of different characteristics. The moon produces elevated illumination of a night-time scene when skies are clear, with a single dominant point source of light in the sky. Sky glow—the reflected light in the atmosphere from anthropogenic sources on the ground (intensified by light reflecting off clouds when present)—similarly increases scene brightness with diffuse illumination from the atmosphere. Glow can be approximated by the calibrated model presented in the 'New World Atlas of Artificial night Sky Brightness' [28], which estimates zenith brightness globally. Light at night may also appear as direct glare sources, which even when not substantially increasing scene brightness can be visible as high-contrast point sources on the visible horizon or closer to mountain lions, dependent on their elevation and distance at the time. The presence of such lights can be best estimated by the upward radiance measured by the Suomi Visible Infrared Imaging Radiometer Suite (VIIRS) sensor [29]. Although the World Atlas of Artificial Night Sky Brightness estimates are derived from the VIIRS data, they diverge in the field, especially at locations near cities, where upward radiance may be low (no development or roads) while sky brightness is high from the nearby development. To date, researchers have not thoroughly sorted out the influence of these two attributes of light at night nor their relationship with and interactions with natural variation in night-time light conditions from the lunar cycle.

We aimed to unravel how ALAN, measured as both sky glow (zenith brightness) and nearby lights (upward radiance), and road proximity affect the habitat selection of a large, generalist top predator like the mountain lion when it faces the most densely populated region within its distribution range. The high human density in Southern California (>20 000 000 people; see [30]) correlates to a large urban development with its associated lights and a high traffic flow on the roads, day and night. Mountain lions in less crowded regions have shown a functional response (i.e. change in selection ratio as a consequence of availability) in which individuals decreased their avoidance of some anthropogenic features when those features became more prevalent on the landscape [16,31]. We expect a negative response to artificial light, highways and minor roads [20,31,32]. We modelled the distribution of GPS-collared mountain lions relative to human-development disturbances, including roads and the two measurements of artificial light at night. The availability of fine-scale tracking data enables the study of individual variability and flexibility in movement as well as habitat-selection patterns [33,34]. We suggest that individual plasticity will have important implications for mountain lions to tolerate different disturbance sources and successfully use human-dominated landscapes. We expect that mountain lions living in anthropized territories decreased their avoidance of some human features, including ALAN [16,31]. In this study, we explored what habitat variables drive habitat selection of mountain lions in Southern California, such as vegetation coverage [16,18,35] or deer prevalence [27,35], and at the same time, we identified individual responses to a) sky glow, b) nearby lights as measured by upward radiance, and c) variation in moonlight.

## 2. Material and methods

### (a) Study area

**Figure 1.**
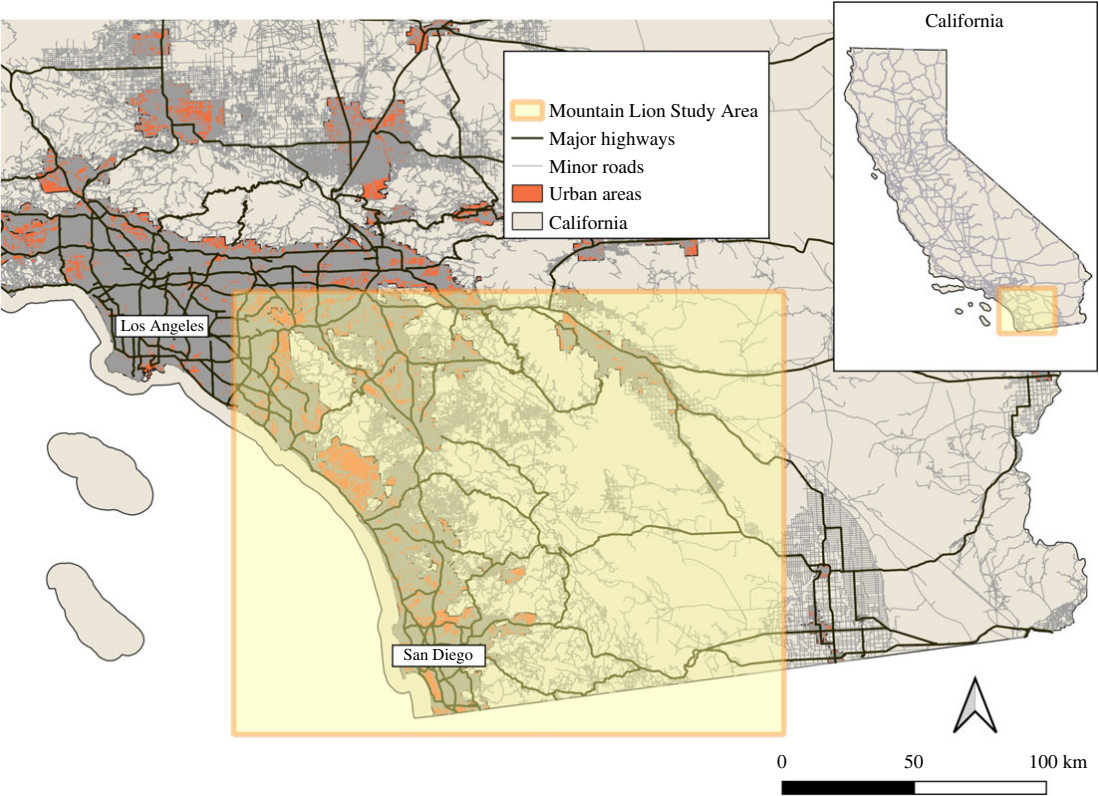
Study area in Southern California including the 102 mountain lion territories. Major highways, minor paved roads and urban areas are represented.

The study area comprises the coastal mountain ranges of Southern California, a region that includes megacities like Los Angeles and San Diego and their corresponding metropolitan areas (figure 1). Mountain lion territories are concentrated in coastal shrub and forest ecoregions and, to a lesser extent, in coastal chaparral or even desert ecoregions [35]. The area experiences a Mediterranean-type climate, with warm summers and cool winters with precipitation in the form of rain.

### (b) Study animals and habitat availability

We used locations of 102 radio-collared mountain lions (49 females and 53 males; 45 adults, >30 months old at trapping, and 57 subadults) monitored in California between 2001–2022. Anesthesia and monitoring protocols were published elsewhere (e.g. [30,35]). Because the aims of those projects were very different, so were the frequencies of locations (i.e. fix rate) they recorded. Thus, to homogenize such variability, we reduced all the frequencies to one location every 2 h (totaling 146 484 mountain lion locations; see electronic supplementary material, table S1). To evaluate the area available to every individual, we studied third-order habitat selection by employing a range distribution approach [36]. For every mountain lion, we calculated the 100% minimum convex polygon (MCP) around its GPS locations in the date range between its initial and final locations (electronic supplementary material, table S1). Then, we applied a 10 km buffer for each of the 102 MCPs as this is the average distance mountain lions travelled in a single monitoring session in a previous study in our study region [37]. Finally, we selected a number of random points in each of these 102 areas (MCP + buffer) proportional to the number of collar points but with a minimum number of 1000 random points per individual (totaling 167 608 random points; see electronic supplementary material, table S1). We assigned a specific time of day to all random locations within the same timeframe as the corresponding individual movement locations, and ensured that all points were situated on land.

### (c) Habitat selection variables

We employed a set of variables to respond to our different questions based on a hypothesis-testing approach for how mountain lions select their home-ranges: 1) *habitat variables* to characterize habitat selection based on findings from previous studies. Land cover and conditions within mountain lion territories are related to foraging opportunities and protection. Previous studies largely agree that mountain lions positively select steeper terrain, shrub and forest cover, and proximity to water, and avoid open habitats and all anthropogenic features like residential areas [16,18,19,27,35,38]. We included: (i) distance to the nearest major highway [39]; and (ii) distance to the nearest minor road [40] (whereas main roads are avoided by mountain lions, likely due to their high traffic flows, secondary roads may be positively selected because they allow movement at a low energetic cost [32,35,37]); (iii) terrain slope; (iv) distance to the nearest shrubland/scrubland (National Land Cover Database (NLCD) categories 51 and 52 from [41]); (v) distance to the nearest forest (NLCD categories 41, 42 and 43); (vi) distance to the nearest grassland (NLCD category 71); (vii) distance to the nearest urban area (NLCD categories 22, 23 and 24); and (viii) distance to the nearest water point (all streams, from [42]). These habitat variables have been shown to determine habitat selection in previous studies, and to provide several resources to this top predator (see e.g. [16,18,20,35]); (ix) mule deer (*Odocoileus hemionus*) based on a habitat suitability model according to the mean expert opinion suitability value for each habitat type for breeding, foraging and cover [43]. When available, mule deer abundance (or its surrogates) was an important explanatory variable because these ungulates are the preferred prey of mountain lions in the western US [27,31,35]. 2) *Light variables*. We focused on light at night because dispersing juvenile lions may avoid lighted areas [44], and mountain lions showed functional response to artificial light in a previous study in which they avoided illuminated areas in dark regions when moving but showed no response in those regions with medium to high exposure [31]. Furthermore, mountain lions hunting in the wildland–urban interface killed deer in the relatively darkest places within the surrounding landscape [31], suggesting these top predators experienced compromised hunting success in those areas with more light exposure. This is especially interesting because Southern California mountain lions included in this study survive in the region that is most exposed to ALAN of its entire range. The exposure to ALAN was defined with two variables: (x) upward radiance from the Suomi NPP Visible and Infrared Imaging Suite (VIIRS), and the zenith brightness, which correlates with overall light exposure (scalar illuminance) more than the VIIRS annual composites [45]. We interpret upward radiance as being correlated with the presence of lights within the pixel (approx. 500 m scale), which should correlate with direct glare within a distance that could influence orientation behaviour, as mountain lions apparently orient toward dark areas and away from city lights [44]. We downloaded the VIIRS day night band (DNB) annual (2014) composite [29] because monthly and daily composites can be challenging, especially when data are missing. We selected the year 2014 because the annual composite VIIRS night-time lights (VNL) version 2.1 provides values from 2012 to 2021, and our mountain lion location data go back to 2002. Thus, we selected a single year—the same year for which data of zenith brightness were available (i.e. 2014; see below). However, our preliminary analyses showed that VIIRS values, although increasing annually, were highly correlated among successive years for a certain locality (i.e. these data are highly temporally autocorrelated, showing a steady pattern; see electronic supplementary material, figure S1). VIIRS light levels were simply extracted from the pixel containing the mountain lion GPS location or randomized point. We also included (xi) the zenith brightness, because it estimates sky glow from light propagating in the atmosphere and not just lights within the pixel. We downloaded these values from the World Atlas of Artificial Night Sky Brightness, a composite product from averaging data from May, June, September, October, November and December from 2014 [28,46]. Finally, other composite that influences the overall illumination is light from natural origins. Thus, we included (xii) natural illumination, which is the combination of moonlight and twilight intensities. We used the moonlit R package (v. 0.9) [47] to estimate natural illumination intensity for any given coordinate and time. This package also allowed us to classify locations as (xiii) day/night locations based on their coordinates and location hour, which allowed us to explore potential differences in habitat selection between day-time and night-time. 3) *Individual traits*. We included (xiv) sex, because previous studies suggest that females, especially when caring for cubs, are more likely to be food-limited than males, and might be tolerant of human presence or attracted to human areas where prey are more abundant and males scarcer, which in turn minimizes infanticide by males [18,38,48]; and (xv) age, because subadults are the dispersing age class [32,44,49].

### (d) Statistical analyses

We first explored the degree of association between all habitat and night light variables with a correlation matrix to measure the amount of multicollinearity. For the subsequent model development, and to minimize multicollinearity among independent variables, we removed distance to the nearest urban area because it was highly correlated with distance to the nearest minor road (see electronic supplementary material, figure S2).

We compared environmental values from mountain lion locations in their home ranges (see above) with different logistic regression mixed models, using the nested effect of the mountain lion's individual identity within the year as random effect to answer the following questions:

(1) Does habitat selection vary between day-time and night-time? In these models, we used all data (both diel periods) and included habitat variables + individual traits + the variable day/night.
(2) Do a) nearby lights (upward radiance), b) sky glow (zenith brightness) and c) natural illumination from moonlight influence mountain lion individual habitat selection at night? In these models, we incorporated all the variables (i.e. habitat variables + light variables + individual traits) but only included night-time locations. To explore inter-individual heterogeneity in illumination variable selection, we allowed for individual random intercepts and slopes [33,50].
(3) Are mountain lions avoiding locations with high local night light (upward radiance) or sky glow (zenith brightness) levels during the day? In these models, we included all the variables but only analysed day-time locations, and we also allowed for individual random intercepts and slopes [33,50].

Before model fitting, all continuous covariates were standardized to mean = 0 and standard deviation = 1. We tested for multicollinearity among predictors with the variance inflation factor (VIF) of each predictor using the HH R package [51] in R [52]. Our analysis suggested that all VIFs were lower than 2, meaning that there was little or no collinearity among input variables.

We used the glmmTMB package [53] and constructed generalized linear mixed models with a binomial error distribution to investigate the effect of the different environmental variables on habitat selection by mountain lions. The general structure of the models is as follows: for the first question we fit two models, consisting of: (1) a full model with all environmental variables with mountain lion identity and year as random effects; and (2) a null model with no fixed effects. For the rest of the questions, we fit four models, consisting of: (1) a full model with all environmental variables (we included mountain lion identity and year as random effects in this model); (2) a full model with all terms, mountain lion identity and year as random effects (additionally we allowed for random slopes and intercepts for light variables (upward radiance and zenith brightness) with fixed intercept variance to account for individual-specific variation in response to light variables [33]); and (3) a model with only light variables, mountain lion identity and year as random effects (additionally we allowed for random slopes and intercepts for light variables). In addition, to explore the effects of weighting the available points, we compared a full model with night data only with the respective weighted logistic regression approach, where the likelihood for the available ‘background’ samples (i.e. y = 0) is weighted with a weight *W* = 1000, while the used points (*y* = 1) keep weight 1 [33]. We compared all models for each subset using Akaike information criterion (AIC). The model with the lowest AIC was considered the most parsimonious and therefore best model. Within models with a ΔAIC < 2, we based our inference on the best-performing model as it appeared to be biologically plausible and relevant [54,55]. We used AIC weight to discuss the covariates supported in the top parsimonious models, i.e. the models within 2 units of the most parsimonious model [56].

We estimated the fit of models in the final set by calculating the marginal coefficient of determination (R2GLMM(m)), which represents the variance explained by fixed factors; and the conditional coefficient of determination (R2GLMM(c)), which represents the variance explained by both fixed and random factors [57]. We calculated the marginal and conditional coefficients of determination in R [52] with the function r2glmm in the MuMIn package [58]. Finally, we assessed the predictive ability of our top models with AUC by randomly partitioning the data for an individual within a year to construct a training set (70% of data) and a test set (30% of data).

## 3. Results

### (a) Does habitat selection vary between day and night?

The full model (including all predictors) performed better in terms of AIC than the null model (electronic supplementary material, table S2, figure S3). Mountain lions positively selected sites with higher deer habitat suitability or proximity to scrubland and forests (electronic supplementary material, table S2, figure S3). Because night/day influenced the probability of mountain lion presence (*p* = 0.003; see electronic supplementary material, table S2, figure S3), we analysed these periods separately (see the following sections).

### (b) Do upward radiance, zenith brightness and moonlight influence mountain lion habitat selection?

**Figure 2.**
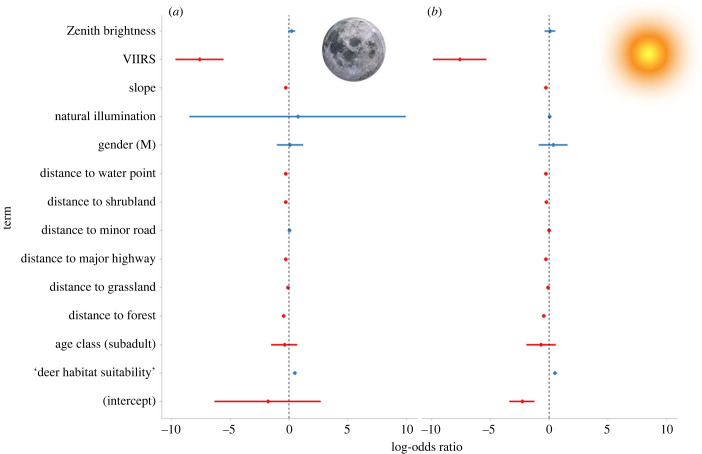
Standardized coefficients from the generalized linear mixed models (GLMM) of the mountain lion habitat selection for (*a*) night and (*b*) day data. Points indicate the mean coefficients, thick bars indicate the 95% credible intervals of the coefficient values. Red and blue line colours indicate negative and positive effects, respectively. The reference level for the categorical variable 'age class' is ‘adult’ and for the sex ‘female’. The overlap of the error bars with the dashed line at zero indicates that the effect of the parameter is not statistically significant (i.e. *p* > 0.05).

The model with the lowest AIC was *Model 2 night*, which contained all of the terms, mountain lion identity and year as random effects, as well as random slopes and intercepts for light (upward radiance and zenith brightness) variables (electronic supplementary material, table S3). Whereas natural illumination did not influence habitat selection, mountain lion locations had substantially lower values of upward radiance than random locations inside their respective territories, and slightly higher levels of zenith brightness (figure 2*a*). Both upward radiance and zenith brightness were included in the best model (electronic supplementary material, table S3), but most of the variance was explained by the upward radiance (log-odds, −7.61), with the probability of mountain lion presence being higher in those locations with lower upward radiance (figure 2*a*). To a much lower extent, at night, the probability of mountain lion presence was higher in areas with higher deer habitat suitability (log-odds, 0.49), close to the forest limit (−0.46) and a shorter distance from the nearest major highway (log-odds, −0.27), whereas distance to the nearest minor road was not significant (log-odds, 0.01; electronic supplementary material, table S3).

**Figure 3.**
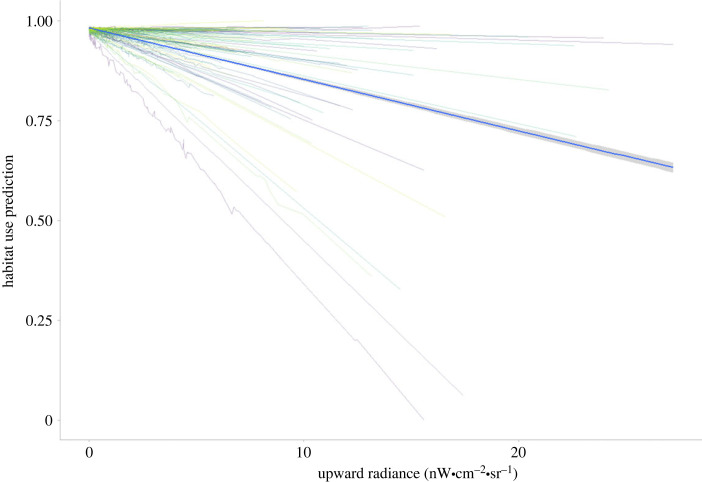
Relationship between the probability of habitat use for individual mountain lions and VIIRS day night band (upward radiance ((nW•cm^–2^•sr^–1^) based on annual 2014 composites). Coloured lines represent the predicted response of each individual, and the blue fitted line with the confidence interval represents the predicted population response from a model with only night data. This figure depicts a full model with all terms, mountain lion identity and year as random effects, and random slopes and intercepts for ALAN variables (VIIRS and zenith brightness).

Interestingly, the model with the lowest AIC included random slopes and intercepts (*Model 2 night*, electronic supplementary material, table S3). This means that individual variability in how mountain lions respond to upward radiance is quite high. The average population response was negative, and every one of the 102 mountain lions responded in a more or less intense way relative to this population response (figure 3; electronic supplementary material, figure S4).

Results were very similar between the unweighted and weighted logistic regression approaches (see electronic supplementary material, table S5). Weighting can introduce a bias unless the use-to-availability ratio is very small [33]. Therefore, weighted alternatives were not further investigated here.

### (c) Are mountain lions also avoiding locations with high night-time light levels during the day?

Again, the model with the lowest AIC built with day-time locations was the one that included all variables, mountain lion identity and year as random effects, and allowed for random slopes and intercepts for upward radiance and zenith brightness (electronic supplementary material, table S4). The most influential variable was again upward radiance (log-odds, −7.59), with higher local night-time glare associated with lower probability of mountain lion presence (figure 2*b*). The slightly elevated probability of presence with higher zenith brightness values that was seen in the night-time data was not apparent during the day. Overall, mountain lions during the day were located in deer-rich habitats (log-odds, 0.51) and close to forests (−0.48) (electronic supplementary material, table S4, figure 2*b*).

## 4. Discussion

Mountain lion habitat selection varied between day and night, and areas highly polluted by nearby artificial light at night (as measured by upward radiance, which is associated with within-pixel light sources more than zenith brightness) were avoided even during the day. Conversely, elevated sky glow was associated with slightly increased probability of presence at night, but not during the day. Despite the existence of a general pattern of avoidance of nearby lights, differences among individuals were large. Habitat features (deer habitat suitability, land use and road network) inside territories also influenced patterns of habitat selection, albeit to a much lesser extent.

Human activities have caused a near-ubiquitous and unprecedented increase in light at night worldwide, and species-specific biological responses to this pollution source will frequently depend on the particular traits of each species [2]. We found a strong mountain lion response to upward radiance compared with zenith brightness, which is consistent with conceptual models that have identified positive and negative phototaxis as being much more closely related to glare than to overall irradiance [2,59]. High radiance values detected by the VIIRS satellite are associated with lights within the immediate vicinity of an animal (within 500 m), while zenith brightness values may be elevated with few nearby lights so long as substantial light sources are within the region (kms), as is in the case in the study area. In this context, it appears that animals are avoiding directly lit zones in the landscape—where they would be able to see actual lamps directly as opposed to sky glow—while continuing to use and in fact slightly preferring habitat with elevated light levels from sky glow. This is consistent with other research on predator–prey relations relative to moonlight and artificial light, wherein some additional illumination benefits the predator [12,60]. To give one example, the common redshank (*Tringa totanus*) switched from foraging by touch to visual foraging both whenever ALAN levels were high and during bright moonlit nights, increasing its foraging time [61]. A recent study based on casual observations has shown that moderated levels of zenith brightness increased the number of mountain lion sightings in Latin American urban areas [24]. However, these moderate light levels may also have enhanced the detectability of mountain lions by humans [62].

Potentially positive responses of mountain lions to elevated sky glow within an overall urbanized landscape contrasts with the negative responses to artificial light at night that are frequently described in the literature. This is, however, understandable when considering the specific attributes of mountain lions, and predators in general. Even within a particular guild, the gradient between positive and negative responses depends on species-specific traits. For instance, the impact of anthropogenic light on European bats depends on the species' foraging guild [63]. The response of bats to lights at foraging grounds separates two groups: 1) those species hunting in open space or right over canopy that exploit insects attracted to streetlights, and consequently benefit from lights [63]; and 2) those species hunting close to or on substrates (i.e. typically forest bats) that are less abundant in illuminated, urbanized landscapes [63].

Mountain lions, whose probability of presence was reduced in locations with higher upward night-time radiance values, are ambush predators that rely upon concealment when hunting deer, their main prey [64,65]. This was the reason given to justify mountain lions’ avoidance of areas with high light levels in a recent study of their habitat selection relative to light at night [31]. Namely, these authors suggested that the likely reason behind these lions' avoidance of areas with high light levels—which they measured as upward radiance—is that this pollution hinders their hunting strategy based on ambushing. Indeed, mountain lions hunting in areas with high night-time light levels killed deer in the relatively darkest places within the landscape [31]. Whereas these authors included only one variable characterizing night-time light (upward radiance), we also included a second one (zenith brightness) and accounted for lunar illumination. Although upward radiance and zenith brightness are partially correlated (electronic supplementary material, figure S2), their biological meaning is slightly different. Upward radiance is related to the presence of lights locally, which would be seen as glare relatively local to the animal's position and therefore could influence orientation, affecting choice of movement direction (see telemetry data reported in [44]). Zenith brightness, in contrast, is more highly correlated with overall brightness (light from all directions, including sky glow; see [45]) and therefore possibly associated with hiding from humans. In all our models, however, the most influential variable by far was upward radiance (although both light variables entered in the best model with zenith brightness giving a positive effect), therefore suggesting that the selection of the light landscape that mountain lions made was more likely based on avoiding human lighting on the horizon or nearer to them, rather than promoting their own local concealment (for which there are studies that provide better approaches; see [31]). Supporting this interpretation, natural illumination (the combination of moonlight and twilight intensities) was not influential at all (see also [31]). We know from unpublished fieldwork (F. Shilling and T. Longcore, 2021) that scalar illuminance (light from all directions, and most likely correlated with zenith brightness) within the study area is well within the range of natural night-time conditions. In a pilot study of the I-15 freeway through the study site, the mean moon-free scalar illuminance at sites several hundred metres away from the freeway was 20.9 ± 10.3 s.d. mlux (*n* = 32), comparable to the illumination from a moon illuminated between 25 and 50% [66]. For species not concerned with predators themselves, we find it consistent that mountain lions would use areas with elevated (but within natural range) light levels from sky glow to forage, but would avoid those places where artificial lights themselves were present (e.g. roads, urban areas and other lit areas) because of the association of lights with the dangers posed by humans. Not coincidentally, when we carried out the analyses with day-only data, locations with high upward radiance (at night) were also avoided, suggesting that artificial illumination may be a signal of overall human activity (roads, commercial, residential areas) and that mountain lions may learn to avoid these areas.

This pattern is generally consistent with previous studies in which mountain lions tended to avoid areas of human activity [16,19,35]. It also mirrors studies of large felids across several human-dominated landscapes around the world that usually avoid human proximity and their associated impacts, including jaguars (*Panthera onca*) in Mexico [67] or Brazil [68], leopards (*P. pardus*) in Kenya [69] or the Eurasian lynx (*Lynx lynx*) in Europe [70], likely because human settlements are important sources of non-natural mortality [30,44,71]. One could expect that generalist species like mountain lions, with a proven capability to develop functional responses to human development, remain unaffected. However, large carnivores patrol large range areas (in our case, MCPs were 430 ± 500 s.d. km^2^ for females and 867 ± 909 s.d. km^2^ for males; see electronic supplementary material, table S1), which inevitably ends up taking these felids to areas where the quality of the habitat is compromised.

The population-level response towards nearby lights was negative. Still, individuals responded with different intensities, likely because not all mountain lions in southern California habitats can select optimal areas with low human disturbance. This variation within a population influences individual strategies and fitness that are crucial for population dynamics, which in turn affects communities and ecosystems [72]. For example, populations that exhibit greater phenotypic variability may be less susceptible to disturbances, better able to colonize new environments and less likely to become locally extirpated. ALAN, a ubiquitous feature of the human footprint, can ultimately be an evolution driver due to the fundamental importance of light to biological systems and the capacity for ALAN to influence multiple processes contributing to individual fitness and even species evolution [73]. To cite some examples, ALAN has affected the developmental pathway (diapause versus direct development) of Mid-European populations of geometrid moths (*Chiasmia clathrata*), suggesting that light pollution may have detrimental effects on insect populations because diapause induction is critical for surviving winter [74]. ALAN could also be behind a global decline of the common glowworm (*Lampyris noctiluca*), a species whose females attract males by glowing, and that did not show an adaptative response to an experimental gradient of ALAN, but delayed or even refrained from glowing [75]. This lack of response is maladaptive for female fitness, as the exposition to ALAN decreased mate attraction success [75]. Nevertheless, other species (or taxa) can take advantage of the new conditions that ALAN provides, as described for brown anoles (*Anolis sagrei*), an invasive urban exploiter [76]. Experimental increased levels of ALAN improved growth, with no apparent costs for stress levels or offspring quality, and with positive effects on reproductive output, altogether suggesting an overall fitness increase [76]. Effects of individual variation are expected to be even more significant in challenging and stressful environments such as urban or human-dominated landscapes [38,77]. A previous study in the same region found that mountain lions killed deer closer than expected to developed areas in those home-ranges containing a lower proportion of developed areas [48]. As noted above, a similar pattern of functional response was found in the less populated Intermountain West, a region dominated by areas with less human development [31].

Among the remaining, notably less influential variables compared to upward night-time radiance, predicted occupancy of mountain lions was higher in those areas with higher mule deer habitat suitability, the main prey of mountain lions in California [48], conditioning this predator's habitat selection [65,78]. Our results of habitat selection at night (i.e. when mountain lions usually move; [44,49]) reflect their selection for routes that maximize low movement costs and good concealment [37,49]. These characteristics in the Mediterranean landscapes of Southern California are usually associated with streams (permanent or not) with abundant vegetation cover, which are preferred by mountain lions during their movement [20,37,44]. Maintaining wide and well-vegetated riparian corridors may be important in maintaining the connectivity of mountain lion subpopulations in disturbed habitats and to ensure the long-term viability of their meta-population system in California. Previous studies demonstrated the importance that very small genetic exchanges can have in increasing the viability of the smallest population units [32,79].

Mountain lions are more likely to be present closer to the major highways at night. This is somewhat in contradiction to previous studies that found that mountain lions avoided any road type in California [20]. However, these approaches to major roads (with cars moving at high speed, rarely stopping) during the night could also be influenced by the fact that deer use road verges when vehicle activity is lower [16], potentially due to a ‘human shield’ effect [80]. Furthermore, studies on other predators in the study area found that highways acted as home range boundaries on territorial individuals, with residents regularly patrolling infrastructure rights-of-way during their periods of activity [81].

Finally, we acknowledge several caveats associated with our study that are important to consider. First, the method we employed for selecting random points within MCP + buffer based on average daily movement may have included some points in our dataset that were not actually available to a mountain lion at a specific location within that range. However, it is important to note that our focus was not on fine-scale movement decisions, but rather on assessing individual variability on higher-order habitat selection within the home range. In terms of ALAN measures, it is worth mentioning that our data correspond to the year 2014, which was the only year for which both upward radiance and zenith brightness data were available. Furthermore, it is crucial to highlight that the World Atlas provides estimates of cloud-free Zenith brightness and does not vary with cloud cover. There are no currently available tools that would allow extrapolation from the World Atlas and (for example) location-specific estimates of low clouds and fog to create an estimate of exposure. Thus, we believe that the fact of not incorporating cloud influences on skyglow does not affect our findings. It is also possible that we are missing important effects at finer scales than those we employed in our study, but gathering such data would require high-performing light meters to be attached to mountain lions, which is not currently feasible. Sky glow does not vary much at distances of 750 m, while upward radiance does, so higher-resolution night light data in lieu of the VIIRS data might have been even more important to habitat selection, were that resolution of data available. Finally, as mentioned earlier, there is still much more work to be done in order to fully comprehend the influence of ALAN on mountain lions. For instance, the impact of ALAN on predation patterns, which has been explored more extensively in other studies [e.g. 31], remains an area that requires further investigation. In our study, our primary goal was to examine how deer abundance determines habitat quality and, consequently, influences mountain lion habitat selection, rather than directly investigating predation patterns themselves.

Overall, mountain lions avoided lit zones in the landscape, which can have cascading effects on both the redistribution of species in the region and the ecosystem functions provided by wildlife (reviewed in [6]). However, these interactions are certainly complex. In the case of the mountain lions themselves, they are top predators that act as providers of resources like carrion for other species as scavengers, while limiting the number of mesopredators through direct predation [82]. Thus, promising avenues of future research are those including the different composites of light pollution—such as light wavelength—to which different species may respond in different ways [2] in complex light landscapes dominated by people.

## Data Availability

Obscured GPS data only are available because the populations of mountain lions are considered sensitive species in the State of California. Code is available from the Zenodo repository: https://doi.org/10.5281/zenodo.7661219 [83]. The data are provided in electronic supplementary material [84].
